# Knowledge and Perceptions about Cervical Cancer and HPV Screening in Women in Rural Areas of Ecuador: A Qualitative Research Study

**DOI:** 10.3390/ijerph191711053

**Published:** 2022-09-03

**Authors:** Estefanía Bautista-Valarezo, Bernardo Vega Crespo, Ruth Maldonado-Rengel, María Elena Espinosa, Vivian Alejandra Neira, Veronique Verhoeven

**Affiliations:** 1 Facultad de Ciencias de la Salud, Universidad Técnica Particular de Loja, Loja 1101608, Ecuador; remaldonado6@utpl.edu.ec (R.M.-R.); meespinosax@utpl.edu.ec (M.E.E.); 2 Facultad de Ciencias Médicas, Universidad de Cuenca, Cuenca 010203, Ecuador; bernardo.vegac@ucuenca.edu.ec (B.V.C.); vivian.neira@ucuenca.edu.ec (V.A.N.); 3 Programa de Doctorado en Ciencias Morfológicas, Universidad de la Frontera, Temuco 4811230, Chile; 4 Programa de Doctorado en Ciencias Médicas, Universidad de la Frontera, Temucho 4811230, Chile; 5 Department of Family Medicine and Population Health, Faculty of Medicine and Health Sciences, University of Antwerp, Universiteitsplein 1, 2610 Antwerp, Belgium; veronique.verhoeven@uantwerpen.be

**Keywords:** rural women, cervical cancer, human papillomavirus, Pap smear, self-test

## Abstract

Background: Cervical cancer continues to be a major health problem in developing countries. Educational programs, as well as Pap and HPV screening and vaccination, are important tools to reduce the morbidity and mortality rates associated with this disease. The objective of this study is to explore the diverse knowledge and perceptions about cervical cancer and the different diagnostic tests for HPV of populations living in the rural parish “El Valle”. Method: A qualitative study was conducted through eight focus groups, which included 46 participants from mixed ethnic groups. A phenomenological analysis was performed. Results: Four topics and seven sub-topics were identified. By analyzing all the narratives, it was possible to identify that the perception of cervical cancer was focused on its severity, secondary to its infectious process and screening periodicity. However, despite the diverse knowledge, indigenous people do not relate it to the human papilloma virus; in addition, there is also certain resistance to undergo the Pap smear test, for reasons such as inaccessibility and its sampling process. Conclusions: It is necessary to develop educational programs for the prevention of cervical cancer and to implement diagnostic alternatives to reach populations with precarious accessibility, as well as women who refuse to undergo the Pap smear test.

## 1. Introduction

According to the data reported by the World Health Organization (GLOBOCAN, 2020), cervical cancer ranks fourth in the world in relation to the tumors affecting women, with a 13.3% incidence and a mortality rate of 7.3 for every 100,000 women [1,2]. In addition, at the global level, it is considered the second most common type of cancer affecting women aged between 20 and 69 years old, with an incidence of 22.8 for every 100,000 women in Latin America. In Ecuador, it has been estimated that nearly 1600 new cases were diagnosed in 2018 [3], a situation that remained unchanged in 2020, with 1534 new cases recorded, thus constituting the second leading cause of malignant neoplasms in women in the country that year [4,5].

In 2020, the estimated incidence of cervical cancer was 604,000 new cases and 342,000 deaths. Of these, 90% corresponded to low- and middle-income countries such as Ecuador [6]. Racial and ethnic minorities, socio-economically disenfranchised, and those in rural areas are affected by discrimination, access to health, and poverty [7]. In addition, these groups have low rates of vaccination, screening, and treatment of cervical cancer, leading to the worst results; for example, in Ecuador, the coverage of Pap smear is low at 54.8% [8].

Cervical cancer implies the presence of an epithelial cell alteration, the origin of which is directly related to infection by some of the human papilloma virus (HPV) serotypes considered high risk (hR)—namely, 16, 18, 31, 33, 35, 39, 45, 51, 52, 56, 58, 59, 68, 73, and 82—which are related to the production of intraepithelial lesions, both pre-malignant and carcinogenic. Serotypes 16 and 18 have been attributed to approximately 80% of the cervical cancer cases [4,8]; for this reason, it is important to identify both the pre-malignant lesions and the infection by these oncoviruses in a timely manner, in order to implement an efficient intervention. This early diagnosis is made by performing HPV and Pap smear tests [9].

In a narrative review on cervical cancer (2021), different techniques approved for the detection of HPV were described. Hybrid capture 2 (HC2) “Identifies with RNA probes of 13 types: 16, 18, 31, 33, 35, 39, 45, 51, 52, 56, 58, 59, 68” and has a high negative predictive value. Polymerase chain reaction (PCR) can identify the most frequent high-, intermediate-, and low-risk HPV types. This test is very sensitive for viral detection [10].

There are several histotypes of cervical cancer, and the prevalence of HPV differs in each of them. In cervical squamous cancer, adenosquamous cancer, and adenocarcinoma of the mucinous intestinal subtype, villoglandular and mucinous signet ring cells are the ones with the highest prevalence of HPV. In contrast, clear cell and serous adenocarcinomas have a lower prevalence of HPV [10].

It is imperative that women from all social strata and from all populations have access to these types of tests, which are oftentimes inaccessible, especially for those who live in rural areas or belong to vulnerable groups, such as indigenous populations and those with limited economic resources [11]. These three factors often go together. It is from this perspective that self-sampling has emerged as an alternative for early detection of the disease. Therefore, the objective of this study is to explore the diverse knowledge and perceptions about uterine cancer and the different diagnostic tests for HPV in rural populations from the rural parish “El Valle” of canton Cuenca of the Azuay province.

## 2. Materials and Methods

The current study followed a qualitative design, and a phenomenological analysis was used. Phenomenology allowed for the describing and interpreting of the reflections and phenomena that arise from life experiences, where a number of either external or internal conditioning factors intervene [12]. Data collection took place through focus groups; the purpose of this technique was to bring to light reactions, attitudes, beliefs, and feelings arising from the participant’s interactions [13]. Focus groups allow one to obtain information and explore ideas that are less accessible to a direct question than in interviews. The discussion of specific topics in a group allowed the participants to remember their own experiences and be strengthened by others. In addition, this instrument, through the focus group guide, allowed for efficient data collection, allowing the user to stimulate the participants and generate a discussion that provided information for research [14].

This paper follows the consolidated criteria for reporting qualitative research (COREQ) guidelines for reporting qualitative research [15].

Ethical approval: The ethical requirements were fulfilled as per the Bioethical Committee of the University of Cuenca, approval code: UC-COBIAS-2020-263. All of the participants and the community gave written informed consent. The participants were free to withdraw from the study at any moment, as well as to refuse to answer the questions. Their names would not be associated with any information they provided. Confidentiality and anonymity of recordings and transcripts were assured throughout the study; the recordings were deleted after transcription was finished. The research was conducted according to the Declaration of Helsinki.

**Sampling**: The participants were selected intentionally by resorting to convenience sampling methods. The subjects who accepted to participate did so voluntarily and with the possibility of withdrawing at any moment, if they wished. All participants were specifically selected for having had an experience of interest to the study, belonging to a rural community, being mixed race, and speaking Spanish. This facilitated the discussion in the focus groups.

**Data collection:** Initially, a thematic guide for the focus groups was prepared. The questions were organized into topics, ranging from general to specific knowledge, and focused on the participants’ points of view (Table 1). On the topic of “New diagnostic tests for papilloma virus”, the researchers explained the instruments (Evalyn brush and a urine collector) for collecting the urine sample and for self-testing to the participants, as they did not have prior experience with this type of test. The focus groups were initiated by providing initial guidance (study objectives) and by expressing commitment to the participants. In addition, their informed consent and permission to audio record the discussions were asked for. The participants were guaranteed confidentiality of their contributions. The focus groups were held with the help of a moderator and an observer, who took field notes. Our research team was multidisciplinary and consisted of MEBV, MEEG, NM, KH, and VV. NM, KH, and VV are GPs and senior researchers at the department of Family Medicine and Population Health of the University of Antwerp (UA), Belgium. All members of the research team have experience in the biomedical health system. Each focus group lasted between 1 and 1.5 h, and at the end of each session, the moderator, the individuals in charge of taking notes, and the lead researcher reported on the key findings and identified any need to further clarify the questions. There were two interviewers—female and male—with experience in qualitative research.

**Analysis:** The data obtained in the focus groups were transcribed, and the research team verified the accuracy of the transcriptions and corrected any errors. All of the field notes and the transcriptions of the focus groups were read for a general understanding of the content. The thematic content analysis, with a phenomenological approach as an interpretive basis of the data, sought to explore the participants’ perceptions, opinions, and interpretations of the research questions [16]. It also involved contracting data from different perspectives and developing categories of meanings [17]. Data saturation was achieved when no new information was obtained from the focus groups or when no new knowledge on the proposed topic came up [18].

The data recorded in the focus groups and the observation notes were transcribed and analyzed using the N-Vivo 11 Pro for Windows software, QSR international, Burlington, MA, USA. A codebook (Table 2) was shared between three researchers, who collectively decided on the description of the dates, until all the codes and quotes were grouped and connected according to broader themes, in order to identify core variables that emerged in a new theory or model of explanation. We identified four main topics, each with one or two pre-defined sub-topics: (1) cervical cancer; (2) papilloma virus (HPV); (3) Pap smear test; and (4) new diagnostic test for HPV. The following section describes the topics and their sub-topics. Quotes were used to enrich the results; they were translated from Spanish to English, while trying to preserve the nuances of the speech (Table 1).

It is important to note that the criteria for assessing the quality of qualitative studies are varied and differ from those applied in quantitative research. It is also essential to ensure the study’s rigor through constant observation, well-structured and executed fieldwork, and capacity for reflection and triangulation among the observers [19,20].

## 3. Results

The final sample comprised of 46 participants aged between 30 and 70. All of them were mestizos, most of them had secondary education, and 2.2% were illiterate. The study participants live in the rural parish “El Valle” of canton Cuenca of the Azuay province (Table 3). Eight focus groups were held, with different participants from different groups. The focus groups lasted between 60 and 90 min, plus 20 min devoted to the initial guidance and commitment to the participants (Table 4).

### 3.1. Cervical Cancer

When defining cervical cancer, most of the participants agreed about severity, malignancy, and fear towards the disease, due to its relationship with death (Q1). Some women related it to the definition of symptoms such as a foul “rotten” smell (Q2), hypogastric pain (abdominal region below the “matrix”; uterus), and vaginal bleeding. Fewer women perceived it in terms of uterine prolapse or “uterus descent” (Q3).

Among the causes of cervical cancer, most of the women agreed upon infectious and contagious diseases, such as those of the urinary tract, venereal diseases, postpartum infections, and untreated or “improperly cured” infections (Q4). A small number of participants also mentioned the following as causes: early initiation of sexual relationships, number of sex partners, and men as mean contagion vectors (Q5). Other causes that were associated, to a lesser extent, were unhealthy diet, use of chemicals to fumigate food, other pathologies such as ovarian cysts, myomas, medications (e.g., contraceptives), and hereditary causes (Q6). All of the participants acknowledged the need to undergo preventive controls and tests, such as the Pap smear, for the prevention of cervical cancer.

***Q1-FG2:*** *I went to the doctor, over there, where they treat women, …. The one from humanitarian help…, there they made me a Pap Smear, and they told me that it was no good, ….. I left devastated from there, the doctor told me “I take the uterus out”. I knew that I was going to die.*

***Q2-FG1:*** *a lady from Loja….with a lot of experience, who taught me how to heal, … She used to tell me, well she didn’t say cancer, she said “it’s getting rotten;” she used to say “chango” “chango”, the uterus is already going “chango” like “pudrid”.*

***Q3-FG2:*** *There are women that, as my mate says, their uterus is about to come out; it turns like a little lemon, a little head already coming out, but my mum usually says that when it is like this, “dear, never leave it like that when you have patients like this; you quickly take the matrix to the inside and make a poultice”.*

***Q4-FG3:*** *Another factor is improperly cured infections, urinary tract infections… I’ve heard about not treating the infections on time and delay it due to fear or embarrassment; it generates complications that can change things in time.*

***Q5-FG2:*** *About sexual relations, about the first relation, very young girls… more risk….. There’s also sexual relations with several people.*

***Q6-FG1:*** *……Well, contraceptive pills can also cause cancer, … I’ve also heard that it can be hereditary.*

### 3.2. Human Papilloma Virus

Although several women associated cervical cancer to infectious diseases, extremely few of them mentioned the human papilloma virus (HPV) as a cause of the disease. Most of the participants mentioned having heard about HPV when vaccination was promoted in the health centers near their homes or through campaigns launched in the schools, where they had to consent to their daughters being vaccinated (Q7, Q8). Knowledge about the vaccine is deficient; however, the study group showed a relatively high acceptance rate towards it. Some women even mentioned the opportunity to have the vaccine now and that their daughters can be vaccinated.

***Q7-FG4:*** *They told me that it was Human Papilloma… That it was transmitted sexually, that when there are infections, let’s say it was like syphilis or gonorrhea.*

***Q-FG7:*** *At school, those who come with the vaccines from the Health Center sent us the authorization for Human Papilloma, they say that it is to avoid infections transmitted by men to women.*

### 3.3. Pap Smear Test

Most of the participants know the Pap smear test as a preventive measure against cervical cancer. They described it as an invasive and painful method that violated their privacy or intimacy (Q9). Although aware of the importance of this test, they acknowledged that they did not undergo it as often as they should, and some women even mentioned never having undergone it. The reasons for such poor acceptance regarding the test were varied; most of the participants stated feeling embarrassment when exposing their body, at the position for sampling, at the pain felt at insertion of the speculum, and at the person collecting the sample being a practicing “student” or a man (Q10). In addition, the participants mentioned how difficult it was to schedule an appointment for the test, or that the results were never delivered to the patients, questioning why they do not have control over appointments to review the result (Q11).

***Q9-FG5:*** *When I did the Pap, I felt pain when they introduced that thing there, not only that… they put it in and say push, push… it’s rough, they tell us to take off our clothes, it looks nasty to me… there’s bleeding sometimes too.*

***Q10-FG3:*** *I have the experience about Paps being performed and there’s always been interns and it’s the people that see you… you’re embarrassed as a woman in this sense, then it’d be something good, ideal, hey, not being exposed so that everyone sees you. The students must learn through the practice and everything, of course; but it’s in fact uncomfortable for a woman.*

***Q11-FG6:*** *Sometimes there’s no chance that they see me for the control, only the days when the doctors come they see us, but we feel embarrassed, it’s traumatic… another thing is that they don’t give us the results, as they only come here one day then we don’t know if we’re OK or not.*

### 3.4. Diagnostic Tests for HPV: Self-Sampling and Urine Tests

When answering this block of questions, all participants indicated being unaware of this type of diagnostic test. Therefore, they were introduced to the topic. The sampling devices were handed over to the participants, and the sample-taking procedures were explained. Subsequently, the questions were focused on the perception of these tests and whether they would be willing to undergo them.

What most drew the attention of all the participants regarding these tests (self-sampling or urine test for HPV) was their speed and the way in which the samples were collected, as they no longer had to expose themselves before the medical personnel. However, most of them believed that, due to the aforementioned characteristics, the test had low reliability, with risks of contamination or improper sampling, and that no in-depth examination would be performed, as compared to the Pap smear test (Q12, Q13).

***Q12-FG7:*** *They tell me that it’s fast, and that my privacy is respected, all the best… But the sample might not be safe, the hygiene care measures, it can get infected, contaminated… the sample might not be taken as it should.*

***Q13-FG4:*** *I’m afraid that the sample is not taken as it should, like my mate says, that they fail to see I’m not OK.*

## 4. Discussion

In order to set up inclusive screening strategies and educational campaigns for cervical cancer, it is important to be aware of prior knowledge and perceptions of diverse groups in society; therefore, we focused specifically on rural women from southern Ecuador. Our findings revealed that, despite being aware of the malignant nature of cervical cancer and the need to periodically undergo the Pap smear test, there was a certain reluctance to this practice. In addition, most of the women were unaware of the relationship between the papilloma virus and cervical cancer, despite knowing about the existence of the vaccine against HPV.

The qualitative approach employed in this study, together with the focus group setting, allowed us to approach the participants and understand their perceptions and knowledge regarding the proposed topic. In addition, as the groups were relatively homogeneous, various nuances in data collection and analysis were deepened and determined through the interactions between the participants [21,22].

In the study group, they defined cancer as a severe disease, as “being rotten”. A study conducted by Shahid, Shaouli; Finn, Lizzie; Bessarab, Dawn; and Sandra C. Thompson, who interviewed 37 indigenous individuals from western Australia, explored the perceptions about cancer, described as “cancer is a synonym for death” by the participants, who even stated their fear towards this disease, which was in line with our study group [23].

Another perception regarding cervical cancer among the indigenous women participating in the study was that they perceived it to be an infectious and contagious disease, with men being the main contagion vectors. Despite this relationship, very few women were aware of the association of HPV as a cause of cervical cancer. This confusion was not only noticed in our study group. In the study of León-Maldonado, Leith et al., conducted with low-income women from Michoacán, México, the authors pointed out women’s confusion about HPV; the women also described it as a contagious disease and attributed it to men’s sexual behaviors [24]. The sexual transmission of HPV implies the recognition of sexual behavior of both men and women as a risk factor for cervical cancer, and promoting safe sex to reduce HPV transmission should be part of an educational strategy [25].

In 2014, the Ecuadorian Public Health Ministry (*Ministerio de Salud Pública*, MSP) incorporated vaccination against HPV in the National Immunization Strategy [26]. This initiative has allowed many places and populations, such as indigenous peoples, to be reached. However, despite this strategy, several women participating in the study were unaware of what HPV was, of its association with cervical cancer, the risk factors, and associated screening methods. The study population perceived the vaccine against HPV as a prevention measure; another way to know about the virus was through the vaccination campaigns. In a systematic review, the objective of which was to explore the knowledge and beliefs of indigenous populations worldwide regarding the vaccine against HPV, it was mentioned that education and easy access to the vaccine improved acceptance in relation to being vaccinated [27].

Cervical cancer screening with the Pap smear test has been one of the main tools of related prevention programs. Screening will continue to be necessary, even after widespread vaccination [28]. In the current study, knowledge about the Pap smear test was good; however, most of the participants mentioned not undergoing the test as often as recommended, and some of them even stated never having undergone it. These data are in line with a retrospective cohort study conducted in Australia by Lisa J. Wop et al., where the participation of indigenous women in cervical cancer screening was studied, observing that they showed lower participation in the screening programs than non-indigenous women [29]. In another study, conducted in Peru by Ferris et al., where a mobile clinic model was implemented, in which women from distant areas could enjoy easier access to Pap smear tests, it was shown that the indigenous women living in isolated areas did not tend to undergo cervical cancer screening tests due to the physical limitation related to reaching the testing sites [30].

New screening tests for HPV are being used, such as the self-sampling and urine tests. Although not yet routinely used, these tests have been validated for use in rural areas in Ecuador [31]. In our study, most of the participants were unaware of this type of test, and the researchers had to explain the topic. After the due explanation, most of the participants concluded that it is a simpler way to find out whether they had the virus or not. However, due to the simplicity of the new tests in sample collection, they simultaneously mistrusted their accuracy when compared to the Pap smear test. These data are in agreement with those presented by Audrey, Muchland et al. in a study conducted in Guatemala that evaluated and compared the acceptability of the HPV self-sampling test among indigenous women, verifying that 81% were comfortable with the test and that 98% were willing to undergo it again [32]. Another study regarding self-sampling for cervical cancer screening, conducted in Argentina with socially vulnerable women, showed that the implementation of this type of test was highly accepted by the population [33].

One of the reflections obtained from the study was the lack of confidence in taking the sample by themselves (self-collection), as it could lead to a wrong diagnosis. In a study carried out on 367 women between 22 and 65 years of age, whose objective was to determine the concordance of the cytological diagnoses made in samples taken by self-collection versus samples collected by doctors, it was determined that there was a good and significant concordance of the diagnoses, with a Kappa value of 0.568 (*p* = 0.040) [34].

Another study on diagnostic tests, conducted on 120 women in rural communities of Cuenca, Ecuador, compared the sensitivity and specificity of clinical sampling versus vaginal and urine self-sampling for HPV diagnosis and found a self-sampling sensitivity of 94.4% (CI 74.2–99.9) and specificity of 92.1 (CI 85.2–95.9), with a negative predictive value of 98.9%; further, urine sampling presented a sensitivity of 88.8% (CI 67.2–96.9) and a specificity of 94.1% (CI 67.2–96.9), with a negative predictive value of 97.6%. In conclusion, self-sampling shows similar sensitivity and specificity when compared to clinical HPV sampling [31].

Cervical cancer prevention programs aim at high detection levels, but accessibility to tests such as the Pap smear is limited for women who live in low- and middle-income countries [35]. Among the main limiting factors regarding access to screening tests are geographical distance issues or the sampling process, as was the case for the Pap smear test; therefore, the role of tests such as self-sampling and urine tests should emerge as an alternative to the classic HPV detection programs.

One of the strengths of this study lies in the fact that the focus groups were homogeneous, which allowed for various interactions in the groups and the discussion of the different ideas, experiences, and opinions. The fact that the participants had no knowledge about self-sampling for HPV diagnosis represents a study limitation, which led the researchers to indicate what the test consisted of and the participants to express their opinions based on this knowledge.

## 5. Conclusions

Our study demonstrated that knowledge about cervical cancer is relatively acceptable in rural women of diverse ethnicity, especially in the aspects related to considering it malignant, its causes, and periodic screening. However, although these topics are relatively clear, factors such as Pap smear sampling, vulnerability to intimacy, lack of follow-up in the results by the health personnel, and limited access to the service have resulted in non-periodicity of the screening procedures.

The need to develop educational programs regarding cervical cancer is a priority in these areas, as well as reinforcing the vaccination programs, which have become bridges for women to know about HPV and how to prevent it. In addition, the incorporation of new, faster, and less-invasive screening methods in promotion and prevention programs is a viable alternative to reach those places and populations where health resources are limited.

## Figures and Tables

**Table 1:** Focus group interview guide.

**Cervical Cancer**
What do you know about cervical cancer?
Do you know the causes of cervical cancer?
Do you know the symptoms of cervical cancer?
**Papilloma Virus**
What do you know about the papilloma virus?
Do you know how the papilloma virus is transmitted?
**Cervical Cancer Diagnosis**
What do you know about the Pap smear test?
Have you ever undergone a Pap smear test?
Which is your experience with the Pap smear test?
**New Diagnostic Tests for the Papilloma Virus**
What do you know about the urine and self-sampling test for the papilloma virus diagnosis?
What do you think about these tests?
Have you undergone any of these tests?

**Table 2:** Topics and sub-topics.

Topics	Subtopics
Cervical cancer	General perceptions Causes of cervical cancer
Papilloma virus (HPV)	General knowledge Forms of infection
Pap smear test	Experiences about Pap smear test sampling
New diagnostic tests for HPV	Ideas about the new diagnostic tests

**Table 3:** Socio-demographic characteristics.

Characteristic	N (%)
**Age (years old)**	
<30	5 (10.9)
30–39	20 (43.5)
40–49	10 (21.7)
50–59	7 (15.2)
>59	4 (8.70)
**Ethnic group**	
Mixed race	46 (100)
**Schooling**	
Illiterate	1 (2.2)
Elementary School	11 (23.9)
High School	30 (64.7)
Higher Education	4 (8.7)

**Table 4:** Focus groups.

Identification Code	Date	Locus	Number of Participants
FG1 *	12 March 2021	Cuenca	9
FG2	18 March 2021	Cuenca	4
FG3	9 April 2021	Cuenca	4
FG4	12 April 2021	Cuenca	3
FG5	24 April 2021	Cuenca	6
FG6	2 May 2021	Cuenca	7
FG7	22 May 2021	Cuenca	4
FG8	11 May 2021	Cuenca	9

* FG: focus group.

## Data Availability

The data sets generated and/or analyzed during the current study are not publicly available, as they contain the sensitive personal information of the participants. The informed consent grants the confidentiality of the participant’s data. However, the data sets are available from the corresponding author upon reasonable request.
